# Proportion of suicides in Denmark attributable to bereavement by the suicide of a first‐degree relative or partner: Nested case–control study

**DOI:** 10.1111/acps.13493

**Published:** 2022-09-13

**Authors:** Alexandra Pitman, Keltie McDonald, Yanakan Logeswaran, Gemma Lewis, Julie Cerel, Annette Erlangsen

**Affiliations:** ^1^ Division of Psychiatry UCL London UK; ^2^ Camden and Islington NHS Foundation Trust St. Pancras Hospital London UK; ^3^ College of Social Work University of Kentucky Lexington Kentucky USA; ^4^ Danish Research Institute for Suicide Prevention Psychiatric Centre Copenhagen Copenhagen Denmark; ^5^ Copenhagen Research Centre for Mental Health, Mental Health Center Copenhagen Mental Health Services Copenhagen Denmark; ^6^ Department of Mental Health Johns Hopkins School of Public Health Hampton Maryland USA; ^7^ Centre for Mental Health Research The Australian National University Canberra Australia

**Keywords:** bereavement, case–control studies, population attributable fraction, registries, suicide

## Abstract

**Objective:**

To provide the first estimates of the risk of suicide after bereavement by the suicide of any first‐degree relative and the proportion of suicides in Denmark attributable to suicide bereavement.

**Methods:**

We conducted a nationwide nested case–control study defining cases as all Danish‐born individuals who died by suicide in Denmark between 01 January 1980 and 31 December 2016 (*n* = 32,248), age‐matched to four living controls. Using three exposure categories (bereavement by the suicide of a relative [parent, offspring, sibling, and spouse/cohabitee]; non‐suicide bereavement; no bereavement) and conditional logistic regression adjusted for pre‐specified covariates we estimated the odds of exposure to suicide bereavement in cases versus controls. We tested whether associations differed for men and women, estimated the population attributable fraction (PAF) of suicides in our population at risk that could be attributed to a first‐degree relative's suicide loss, and estimated the attributable fraction among the exposed (AFe).

**Results:**

Suicide bereavement was associated with an increased odds of suicide when compared with no bereavement (OR_adj2_ = 2.90, 95% CI: 2.46–3.40) or non‐suicide bereavement (OR_adj2_ = 1.48, 95% CI: 1.25–1.74). There was no evidence to support any interaction with sex. PAF (0.69%; 95% CI: 0.62%–0.77%) and AFe (60.12%; 95% CI: 53.19%–66.03%) estimates suggested that in Denmark 0.69% of suicides, and 60% of suicides among suicide‐bereaved relatives, could be prevented if it was possible to address all factors increasing suicide risk in suicide‐bereaved relatives.

**Conclusion:**

Suicide bereavement in relatives and partners contributes to at least one in 145 suicides in Denmark.

**SIGNIFICANT OUTCOMES**

- Risk of suicide is elevated in relatives and partners bereaved by suicide, whether compared with people bereaved by other causes or people unexposed to bereavement.
- An estimated one in 145 suicides in Denmark is attributable to the suicide loss of a first‐degree relative.
- If we could eliminate exposure to suicide bereavement (or address factors increasing suicide risk in Danish people bereaved by a relative or partner's suicide), we could prevent an estimated 60% of suicides in this group.

**LIMITATIONS**

- Our exposure classification under‐ascertained bereavements recorded i) prior to 1980, ii) overseas, iii) of relatives not identified by linkage prior to 1953, or iv) where individuals had been exposed to both suicide bereavements and other bereavements.
- Our analyses underestimated psychopathology because of the lack of data on primary care presentations and outpatient care, as well as episodes of mental illness or self‐harm in which no healthcare presentations were made.

## 1 INTRODUCTION

Suicide bereavement is estimated to affect 22% of the population across the life course and 4% of the population in any given year.^1^ While most are able to process the loss, suicide bereavement is also associated with an elevated risk of suicide, suicide attempt and psychiatric illness.^2^, ^3^ Up to one in 10 people bereaved by the suicide of a relative or friend make a subsequent suicide attempt.^4^ Providing support for people bereaved by suicide features in many international suicide prevention strategies^5^ with the intention of relieving distress and mitigating risk of suicide. However, there exists no estimate of suicide risk after the suicide of any first‐degree relative, including partners, nor an estimate of the population attributable fraction (PAF) of suicides attributable to suicide bereavement or the attributable fraction among the exposed (AFe). The importance of a PAF estimate lies in conveying a sense of the public health impact of the exposure (suicide bereavement) on the outcome (suicide mortality). It does this by estimating the proportion of suicides in a population at risk that would be prevented if there had been no exposure to suicide bereavement of a first‐degree relative (or if we could address all factors increasing suicide risk in suicide‐bereaved relatives). The complementary AFe estimate conveys the proportion of suicides among the suicide‐bereaved that can be attributed to their exposure to suicide loss.

Systematic reviews of this literature show that the risk of suicide after suicide loss has only been quantified in specific kinship groups bereaved by suicide.^2^, ^6^, ^7^ Population‐based studies describe excess suicide risk among parents bereaved by an adult child's suicide compared with non‐bereaved parents,^8^ among offspring bereaved by parental suicide compared with parental loss by non‐suicide causes,^9^ and among suicide‐bereaved spouses whether compared with non‐bereaved spouses or spouses bereaved by other causes.^3^ However, these studies had limited power to investigate sex differences. In filling a key evidence gap, we hypothesised that the risk of suicide would be higher in individuals bereaved by the suicide of any first‐degree relative than individuals bereaved by other causes of death and non‐bereaved individuals, and that the relationship would be strongest when compared with non‐bereaved individuals.

### 1.1 Aims of the study

- To use Danish population‐based registry data to estimate the association between bereavement by the suicide of any identified first‐degree relative (parents, children, siblings, and partners/spouses linked by family, marriage or cohabitation status using a household variable) and risk of suicide in relation to two reference groups: (a) people bereaved by other causes of death (controlling for the trauma of bereavement per se) and (b) people not bereaved by any identified first‐degree relative's death.
- To assess whether the relationship differed for men and women.
- To estimate the proportion of suicides attributable to suicide bereavement in the population (PAF) and among those exposed (AFe).

## 2 METHODS

### 2.1 Study design

We conducted a population‐based nested case–control study (case–control within a cohort study) to quantify the risk of suicide after exposure to a relative's suicide, whether related genetically (parents/children/siblings), by adoption, or partnership (spouse/civil partner/cohabiting partner), with no age restrictions. Cases were defined as people who had died by suicide (our outcome), controls were age‐matched individuals alive on the day of suicide, and our exposure of interest was prior death of a linked first‐degree relative since 1980.

### 2.2 Study sample

An individual‐level data linkage on all Danish‐born residents who had not migrated since birth was conducted using a unique personal identification number^10^ across the following population registers: the Civil Registration System (since 1968),^11^ the Register of Causes of Death (since 1970),^12^ the Registry of Social Pension and Income (since 1980),^13^ the Psychiatric Central Research Register(since 1969),^14^ and the National Patient Register(since 1977).^15^ Cases were defined as all individuals who died by suicide in Denmark between 01/01/1980 and 31/12/2016 (n = 32,248), based on the relevant codes from the International Classification of Disease [ICD] 8th and 10th revisions (see Tables 1 and S1).^16^, ^17^ We defined 1980 as the beginning of our observation period to provide us with over 10 years of previous data on psychiatric confounders.

**TABLE 1:** Socio‐demographic and clinical characteristics of main sample (*N* = 147,154)

Characteristic^a^	Cases^b^ (*N* = 29,513)						Controls^b^ (*N* = 117,641)					
	*N*			%			*N*			%		
*Sex* ^c^												
Male	20,011			67.8			56,820			48.3		
Female	9502			32.2			60,821			51.7		
Age at time of matching (median, IQR)	52			40–66			52			40–66		

	Exposed to suicide bereavement^j^ (*N* = 340, 1.2%)		Exposed to other bereavement (*N* = 4,478, 15.2%)		Unexposed (*N* = 24,695, 83.7%)		Exposed to suicide bereavement^j^ (*N* = 504, 0.4%)		Exposed to other bereavement (*N* = 12,669, 10.8%)		Unexposed (*N* = 104,468, 88.8%)	
	*N*	%	*N*	%	*N*	%	*N*	%	*N*	%	*N*	%
*Sex* ^c^												
Male	213	62.7	2,660	59.4	17,138	69.4	203	40.3	4,225	33.4	52,392	50.2
Female	127	37.4	1,818	40.6	7,557	30.6	301	59.7	8,444	66.7	52,076	49.8
Age at time of matching (median, IQR)	48	35–62	67	49–78	50	39–63	50	39–63	69	50–79	51	39–64
Age at exposure (median, IQR)	40	22–54	59	40–68	44	32–57	39	24–51	58	40–68	45	32–58
*Household income level (quartiles)* ^c^												
1 (lowest)	48	14.1	556	12.4	3,266	13.2	72	14.3	1,546	12.2	11,147	10.7
2	110	32.4	1,857	41.5	7,638	30.9	113	22.4	4,993	39.4	26,626	25.5
3	96	28.2	1,300	29.0	7,281	29.5	163	32.3	3,384	26.7	31,412	30.1
4 (highest)	86	25.3	765	17.1	6,510	26.4	156	31.0	2,746	21.7	35,283	33.8
*Marital status* ^d^												
Never married	139	40.9	965	21.6	8,474	34.3	195	38.7	2,432	19.2	28,562	27.3
Married/registered partnership	142	41.8	3,060	68.3	11,433	46.3	250	49.6	9,248	73.0	62,174	59.5
Divorced/dissolved partnership	54	15.9	393	8.8	3,531	14.3	55	10.9	845	6.7	8,156	7.8
Widowed/bereaved after partnership	5	1.5	60	1.3	1,257	5.1	4	0.8	144	1.1	5,576	5.3
*Previous self‐harm* ^e^	41	12.1	325	7.3	1,980	8.0	11	2.2	108	0.9	690	0.7
*Mental health conditions* ^f^												
Any	72	21.2	733	16.4	5,819	23.6	24	4.8	471	3.7	3,471	3.3
PTSD	<3	–	<3	–	14	0.1	<3	–	<3	–	7	<0.1
Depression	23	6.8	309	6.9	2,339	9.5	4	0.8	204	1.6	1,180	1.1
Anxiety	5	1.5	46	1.0	398	1.6	3	0.6	45	0.4	344	0.3
Substance/alcohol use	45	13.2	402	9.0	3,083	12.5	13	2.6	224	1.8	1,723	1.7
Severe mental illness	27	7.9	217	4.9	1,962	7.9	8	1.6	100	0.8	948	0.9
*Physical health conditions* ^g^												
Any	17	5.0	324	7.2	1,173	4.8	12	2.4	604	4.8	3,634	3.5
Cardiovascular disease	7	2.1	181	4.0	588	2.4	8	1.6	361	2.9	1,985	1.9
Hypertension	<3	–	46	1.0	205	0.8	3	0.6	104	0.8	677	0.6
Diabetes mellitus	4	1.2	49	1.1	261	1.1	<3	–	110	0.9	742	0.7
COPD	4	1.2	69	1.6	206	0.8	<3	–	70	0.6	472	0.5
Relationship to the deceased												
Child	26	7.7	156	3.5	–	–	39	7.7	564	4.5	–	–
Parent	87	25.6	796	17.8	–	–	119	23.6	2,192	17.3	–	–
Partner	192	56.5	3,455	77.2	–	–	309	61.3	9,706	77.6	–	–
Sibling	35	10.3	71	1.6	–	–	37	7.3	207	1.6	–	–
*Living with deceased at time of death* ^h^												
Yes	160	47.1	2,986	66.7	–	–	243	48.2	8,437	66.6	–	–
No	167	49.1	1,382	30.9	–	–	248	49.2	3,876	30.6	–	–
Time elapsed since split for ex‐partners (median, IQR)	4	2–7	5	2–12	–	–	4	1–9	6	2–12	–	–
*Number of total bereavements* ^i^												
1 (suicide)	292	85.9	–	–	–	–	440	87.3	–	–	–	–
1 (other bereavement)	–	–	4,274	95.4	–	–	–	–	12,164	96.0	–	–
>1 (suicide solely)	6	1.8	–	–	–	–	<3	–	–	–	–	–
>1 (other bereavement solely)	–	–	204	4.6	–	–	–	–	505	4.0	–	–
>1 (suicide and other bereavement)	42	12.4	–	–	–	–	63	12.5	–	–	–	–

Abbreviations: COPD, chronic pulmonary obstructive disease; IQR, interquartile range; PTSD, post‐traumatic stress disorder.

^a^ Values are frequencies and percentages unless otherwise specified. Values for each time‐varying covariate were measured at the same timepoint for all individuals within each set of that in any set of one case and four controls, based on the earliest date of exposure within each set.

^b^ Cases were individuals who died by suicide, based on the following codes in the Register of Causes of Death: ICD‐8 codes: E950‐E959 or where or where manner of death was recorded as "‘suicide’; ICD‐10 codes: X60‐X84 or where or where manner of death was recorded as ‘suicide’. Cases and controls were matched by birth year on age at suicide/age at matching.

^c^ Identified in the Registry of Social Pension and Income, and calculated as the total income within the household divided by the total number of adults living in the household, then categorised into quartiles based on the national annual income averages for that year in Denmark. For children (aged under 18 years), income was derived from the household income of the highest‐earning parent.

^d^ Identified in the Civil Registration System.

^e^ Identified from inpatient psychiatric or medical admission for self‐harm or where the reason for contact was recorded as self‐harm.

^f^ Identified from inpatient psychiatric admission recording a diagnosis of specific psychiatric disorders.

^g^ Identified from inpatient medical admission recording a diagnosis of specific physical health conditions.

^h^ Missing data on this variable for cases were: exposed to suicide bereavement: 13, 3.8%; exposed to other bereavement: 110, 2.5%; missing data for controls were: exposed to suicide bereavement: 13, 2.6%; exposed to other bereavement: 356, 2.8%.

^i^ Note that only the first bereavement was counted for the purposes of covariate measurement.

^j^ Any individual bereaved by both suicide and non suicide‐death was classified as suicide‐bereaved whether the suicide death pre‐ or post‐dated the non‐suicide death. Overall, in the full sample 0.6% were exposed to suicide bereavement, 11.7% were exposed to other bereavement, and 78.8% were unexposed.

Using incidence density sampling,^18^ we matched each case to four controls born in the same year and alive on the date of each case's suicide (the matching date). To reduce computational burden, controls were drawn from a random 25% sample of the total Danish population as per precedent.^19^, ^20^

### 2.3 Measures

#### 2.3.1 Exposure

Exposure was classified according to bereavement by the death of a parent, child, sibling, partner or former partner between 01 January 1980 and 31 December 2016 that occurred prior to the suicide of the case (or corresponding matching date among controls), using relevant ICD‐8 and ICD‐10 codes (Table S1). We defined three categories of exposure: bereavement by a relative's suicide, bereavement by a relative's non‐suicide death and no bereavement. Any individual bereaved by both suicide and non‐suicide death was classified as suicide‐bereaved whether the suicide death pre‐ or post‐dated the non‐suicide death. To ascertain these, cases and controls were linked with their kinship groups (parents/children/siblings/partners) using information on family type, partners, and household identification number as recorded in the Civil Registration System. We included biological, step, and adoptive parents/children/siblings. We defined partnership as spouses or civil partners (opposite and same‐sex) and cohabiting couples, using a standard proxy for cohabiting couples (see Supplemental Methods S1), as per precedent.^3^, ^20^ This identifies cohabiting couples as the only two opposite‐sex adults living in the same household who are not genetically related, with an age difference less than 15 years. Individuals with no relatives were, by default, classified as unexposed. However, some born before 1953 may have had parents/siblings unlinked to them because of the timing of personal identification number assignment from 1968^10^ (see Supplemental Methods S1).

#### 2.3.2 Confounders

Seven confounders were identified a priori based on the literature, identifying pre‐bereavement differences on physical and mental health measures between people bereaved by suicide and bereaved or non‐bereaved controls.^2^ Confounders were: sex (male; female); marital status (never married; married/registered partnership; widowed/bereaved; divorced/separated); family size; household income level (quartiles); any pre‐bereavement history of self‐harm recorded on inpatient psychiatric or medical admission; any pre‐bereavement history of depression, post‐traumatic stress disorder (PTSD), anxiety disorder, substance/alcohol use, or severe mental illness recorded on inpatient psychiatric admission; and any pre‐bereavement history of specific physical health conditions (hypertension, chronic obstructive pulmonary disease, cardiovascular disease, diabetes mellitus) recorded on inpatient medical admission.

Marital status was used as distinct from cohabitation status to capture the confounding effect of divorce.^21^ Our derived variable for family size reflected the number of parents/children/siblings/partners alive and linked to each case/control at any point prior to the index bereavement, accounting for the greater risk of bereavement in those with larger families and also their greater connectedness.

All covariates were updated on the exact date of change (e.g. hospital admission) or (for marital status and income) January 1 each calendar year. Within each set of one case and four controls we measured values for each time‐varying covariate at the same timepoint, based on the earliest date of exposure within each set. For individuals who had experienced multiple bereavements, covariates were measured for the first bereavement. For sets in which no‐one was exposed, we assigned a pseudo index date^22^ (see Supplemental Methods S1).

### 2.4 Statistical analysis

We conducted our main analyses on cases (who had died by suicide) and living age‐matched controls who had complete data on all variables in the analysis, comparing their exposure to suicide bereavement, other bereavement, and no bereavement. As both cases and exposures were defined according to a suicide, any individual who died by suicide between 1980 and 2016 was classified as a case, but would also have defined their relatives as exposed to suicide bereavement. The demographic and clinical characteristics of cases and controls were examined using frequencies/percentages (categorical variables) or medians/interquartile range (continuous variables). We estimated the association between suicide bereavement (exposure) and odds of suicide using conditional logistic regression, with each case forming a separate stratum. As controls were randomly selected from the appropriate risk sets, estimated odds ratios were indicative of unbiased estimates of the corresponding incidence rate ratios from the wider cohort.^23^

Initially, we estimated the univariable association between bereavement status and suicide, changing the reference category to compare suicide bereavement with (a) other bereavement and then (b) no bereavement. We re‐ran models adjusted for potential confounders (sex, marital status, family size, income, pre‐bereavement self‐harm, pre‐bereavement psychiatric illness, pre‐bereavement physical illness). We used Wald tests to assess effect modification by sex within each model including an interaction term between bereavement and sex.

We estimated the PAF and AFe for our univariable and multivariable models, with 95% confidence intervals, using the user‐written *punafcc* command in Stata.^24^ To estimate these parameters, we re‐fitted the univariable and multivariable conditional logistic regression models to compare the risk of suicide in the suicide‐bereaved with a combined reference group of other bereaved and non‐bereaved individuals.

To establish whether missing data were associated with suicide bereavement (exposure) and/or with suicide (outcome), we compared characteristics between the analytic sample and those excluded because of missing information. A proportion of data were missing because income and marital status data were only available from 1980, therefore pre‐bereavement income and marital status were unavailable for some individuals who were bereaved early on in the follow‐up period.

We carried out a sensitivity analysis to estimate associations using multiply imputed data, assuming data were missing at random. We performed multiple imputation by chained equations based on 50 iterations, including all confounding variables, exposure, and outcome. To account for the matched design, we included the matching variable (birth year) within our imputation model.^25^ We also added date of confounder assessment (bereavement date in exposed, matching date for unexposed) as an auxiliary variable in the imputation model given its strong association with missingness within our sample. We re‐ran analyses on the imputed dataset, combining estimates using Rubin's rules.

Data preparation was carried out in SAS software version 9.4.^26^ Data analysis was carried out in Stata 16 software.^27^

## 3 RESULTS

Within the total sample of eligible participants (N = 161,240), there were 12,981 (8.1%; cases: *N* = 2734, 8.5%; controls: *N* = 10,247, 7.9%) with missing information on income or marital status. Given the matched design, this excluded 14,086 (8.7%) individuals. These individuals were older and more likely to be female, widowed, and to have a history of depression, or any physical disorder (Table S2).

Our main analytic sample (*N* = 147,154) with complete data consisted of 29,513 cases who died by suicide and 117,641 living age‐matched controls (Table 1). Cases were more likely to be male, within the lowest income quartile, divorced or never married, and to have a history of self‐harm or any psychiatric admission, but less likely to have a history of admission for a physical health disorder.

Suicide bereavement was associated with increased odds of suicide compared with exposure to other bereavement and with no bereavement (Table 2). In unadjusted analyses, and (with slight attenuation) when adjusted for all confounders, the association between suicide bereavement and risk of suicide was stronger when the reference category was no bereavement (OR_crude_ = 2.98, 95% CI: 2.59–3.42; OR_adj2_ = 2.90, 95% CI: 2.46–3.40) than non‐suicide bereavement (OR_crude_ = 1.82, 95% CI: 1.58–2.10; OR_adj2_ = 1.48, 95% CI: 1.25–1.74).

**TABLE 2:** Risk of suicide in suicide‐bereaved individuals compared with two comparison groups (other bereaved and non‐bereaved)

		Unadjusted		Adjusted 1^a^		Adjusted 2^b^	
Exposure group	Case/control	OR	95% CI	OR	95% CI	OR	95% CI
*Non‐bereaved as reference category*							
Non‐bereaved	24,695/104,468	1.00	–	1.00	–	1.00	–
Suicide‐bereaved	340/504	2.98	2.59–3.42	3.15	2.70–3.67	2.90	2.46–3.40
*Other bereaved as reference category*							
Other bereaved	4478/12,669	1.00	–	1.00	–	1.00	–
Suicide‐bereaved	340/504	1.82	1.58–2.10	1.56	1.34–1.83	1.48	1.25–1.74

Abbreviations: CI, confidence interval; OR, odds ratio.

^a^ Adjusted for sex, marital status, family size, household income level.

^b^ Adjusted for all variables in adjustment 1, plus pre‐bereavement history of self‐harm, mental and physical health conditions.

We found no evidence to support an interaction with sex, whether comparing suicide bereavement with no bereavement (*p* = 0.107), or non‐suicide bereavement (*p* = 0.896)(Table 3).

**TABLE 3:** Risk of suicide in suicide‐bereaved individuals compared with two comparison groups (other bereaved and non‐bereaved), stratified by sex

			Unadjusted			Adjusted 1^a^			Adjusted 2^b^		
Exposure group	Stratum	Case/control	OR	95% CI	*p*‐Value for interaction test^c^	OR	95% CI	*p*‐Value for interaction test^c^	OR	95% CI	*p*‐Value for interaction test^c^
*Non‐bereaved as reference category*											
Non‐bereaved^d^	Male	17,138/52,392	1.00	–	–	1.00	–	–	1.00	–	–
	Female	7557/52,076	1.00	–	–	1.00	–	–	1.00	–	–
Suicide bereaved^e^	Male	213/203	3.41	2.81–4.15	0.517	3.23	2.62–3.98	0.710	3.26	2.62–4.06	0.107
	Female	127/301	3.11	2.52–3.83		3.05	2.44–3.81		2.50	1.96–3.18	
*Other bereaved as reference category*											
Other bereaved^f^	Male	2660/4225	1.00	–	–	1.00	–	–	1.00	–	–
	Female	1818/8444	1.00	–	–	1.00	–	–	1.00	–	–
Suicide bereaved^e^	Male	213/203	1.62	1.32–1.98	0.238	1.43	1.15–1.77	0.244	1.49	1.19–1.86	0.896
	Female	127/301	1.93	1.56–2.40		1.72	1.37–2.16		1.45	1.13–1.86	

Abbreviations: CI, confidence interval; OR, odds ratio.

^a^ Adjusted for sex, marital status, family size, household income level.

^b^ Adjusted for all variables in adjustment 1, plus, pre‐bereavement history of self‐harm, mental and physical health conditions.

^c^ P‐value from Wald test for interaction between exposure and sex.

^d^ The suicide bereaved group comprised 49% men and 51% women.

^e^ The non‐bereaved group comprised 54% men and 46% women.

^f^ The other bereaved group comprised 40% men and 60% women.

Our PAF estimate, based on the fully adjusted model, suggested that approximately 0.69% (95%CI: 0.62%–0.77%) of suicides could be prevented in the Danish‐born population of Denmark if no first‐degree relatives experienced suicide bereavement, assuming a causal association (Table 4). The corresponding AFe estimate (60.12%; 95%CI: 53.19%–66.03%), suggested that if we could identify and remove all factors that increased suicide risk in people bereaved by a relative or partner's suicide, we could prevent 60% of suicides among Danish‐born individuals bereaved by the suicide of a first‐degree relative (Table 4).

**TABLE 4:** Attributable impact of suicide bereavement on risk of suicide in the population at‐risk

	OR (95% CI)	PAF % (95% CI)	AFe % (95% CI)
Unadjusted	1.00 (0.86–1.14)	0.73 (0.67–0.79)	63.30 (57.84–68.05)
Adjusted 1^a^	1.00 (0.85–1.16)	0.73 (0.67–0.79)	63.35 (57.36–68.50)
Adjusted 2^b^	2.51 (2.14–2.94)	0.69 (0.62–0.77)^c^	60.12 (53.19–66.03)

Abbreviations: 95% CI, 95% confidence interval; AFe, attributable fraction among the exposed; OR, odds ratio; PAF, population attributable fraction.

^a^ Adjusted for sex, marital status, family size, household income level.

^b^ Adjusted for all variables in adjustment 1, plus pre‐bereavement history of self‐harm, mental and physical health conditions.

^c^ 0.69% is equivalent to one in 145 (indicating that suicide bereavement contributes to one in 145 suicides in Denmark). Based on the 29,513 cases of suicide we identified in Denmark over the period 1980 to 2016, an estimated 204 suicides (0.69% of 29,513) could be attributed to suicide bereavement by a first‐degree relative over this period.

Our findings were robust to a sensitivity analysis based on the multiply imputed sample (Tables S3 and S4).

## 4 DISCUSSION

### 4.1 Main findings

We found, in our nested case–control study of the whole Danish population, an elevated risk of suicide in relatives and partners bereaved by suicide, whether compared with people bereaved by other causes or people unexposed to bereavement. This is the first study to have quantified the suicide risk associated with suicide bereavement for all first‐degree relatives (including partners), complementing previous studies presenting risk estimates for individual kinship groups.^3^, ^8^, ^9^ Our study is also the first to have measured the population effect of exposure to suicide bereavement, describing the overall public health burden of suicide loss by providing a PAF estimate that 0.69% of suicides in Denmark would be prevented if the exposure were removed (or if we could address all factors that increased suicide risk after this exposure), assuming causality. The contribution of suicide bereavement to population suicide risk is therefore lower than for other risk factors such as PTSD (1.6%),^28^ self‐harm (7.2%),^29^ unemployment (10%)^29^ or childhood sexual abuse (11.3%).^30^

Our hypothesis was supported that the magnitude of risk associated with suicide bereavement was greater when compared with unexposed controls than bereaved controls, in keeping with our broader understanding of the impact of suicide bereavement relative to other losses.^2^ However, we found no sex differences in the magnitude of risk, in the context of having greater statistical power than other studies investigating sex differences in such associations through our inclusion of all kinships. Our reliance on in‐patient data to identify psychiatric and physical health diagnoses would tend to focus on the most severe episodes requiring hospitalisation, reducing the effects of differential help‐seeking. Generally, our use of large population‐based data and a priori selection of confounders minimises the risk of chance, bias and confounding as explanations for these findings.

### 4.2 Findings in the context of other studies

No other studies investigating the effects of suicide bereavement have calculated PAF/AFe estimates, and few examples are provided in the research literature for other suicide risk factors. We are therefore limited in the comparisons we can make to those calculated for other risk factors, such as sexual abuse or PTSD, although our estimate remains much lower than these. Our estimate of the magnitude of suicide risk associated with suicide bereavement across a range of kinship groups complements evidence from registry‐based studies describing an elevated risk of suicide in individual kinship groups; after the suicide of an adult child,^8^ a child of any age,^20^ a parent,^9^ a sibling,^31^, ^32^ or of a partner^3^, ^20^, ^33^ whether defined as a cohabitee or legal spouse.^20^, ^34^ Our finding that the magnitude of suicide risk did not differ by sex (for all kinship groups) is striking given men's lesser likelihood of help‐seeking for low mood,^35^ reducing opportunities for intervention. However, these findings reflect combinations of heterogeneous sex differences within different kinship groups as described in previous literature.^3^, ^8^, ^9^ In other analyses, the magnitude of suicide risk is reported to be greater in female spouses bereaved by suicide than male spouses,^3^ in suicide‐bereaved mothers than fathers,^8^ in female offspring bereaved by maternal suicide than male offspring,^9^ and in male offspring bereaved by paternal suicide than female offspring,^9^ although none of these studies provided evidence to support statistically significant gender differences. It is possible that the findings of our interaction tests may reflect joint modification by sex and kinship group, and that appropriately powered kinship‐specific analyses may be more easily interpretable.

### 4.3 Strengths and limitations

Providing the first estimate of suicide risk associated with suicide bereavement for all first‐degree relatives, including partners, was possible using a case–control design and data on an entire population at risk.^36^ In using a representative population‐based sample, we identified all first‐degree relatives bereaved by suicide in Denmark since 1980, with no loss to follow‐up. The Danish registers are known to be reliable for psychiatric research because they provide completed records of all mental health conditions (including self‐harm) diagnosed during inpatient episodes from 1969, as well as data on cause of death from 1970, all physical health conditions diagnosed during inpatient episodes from 1977, and data on income, migration and marital status from 1980. Reliability of suicide classification across Denmark is judged to be strong compared with other Scandinavian registers,^37^ although validity does rely on the physicians certifying deaths, which may be subject to individual differences and secular changes.^12^

The nested case–control design, matching cases and controls on birth year, partially dealt with the problems encountered using a cohort study design in addressing heterogeneity of kinship groups and age at loss (because individuals in each set were the same age). Our use of routine registry data addressed problems of recall bias typical of cross‐sectional data, and meant we had low levels of missing data and for only two key variables (income and marital status). Our exclusion of some (8.7%) cases and controls from the analysis because of missing data may have biased our findings based on their characteristics. However, our findings were robust to investigation of the effects of potential biases introduced by missing data. Our exposure classification under‐ascertained bereavements recorded prior to 1980, overseas, of relatives not identified by linkage prior to 1953, or where individuals had been exposed to both suicide bereavements and other bereavements. We did not take into account multiple exposures (of the same or another bereavement type) because we wanted to investigate the specific impact of suicide loss, and this was not a clear confounder. Our use of a household variable to capture cohabitees as well as legally‐recognised partnerships addressed under‐ascertainment of partner bereavement, but the standard partner definition excluded same‐sex cohabitees and couples with larger age differences.

Our use of variables from a range of linked population registers meant we were able to adjust not only for pre‐bereavement psychopathology, but also other important potential sociodemographic and clinical confounders. This improves on previous registry‐based analyses taking into account only age, sex, time since loss and kinship.^32^ However, like any observational study, we cannot exclude the possibility of residual confounding. Despite the many advantages of registry data in overcoming biases, particularly recall bias, they lack subjective measures such as relationship quality, as well as episodes of physical and mental illness unrecognised during hospital admission. However, our direct comparison of bereavement by suicide, non‐suicide bereavement and non‐bereaved controls accounted for the trauma of bereavement per se. Our analyses underestimated psychopathology because of the lack of data on primary care presentations and outpatient care, as well as episodes of mental illness or self‐harm in which no healthcare presentations were made. Our PAF estimate may have underestimated the impact of suicide loss prior to 1980, as we only had complete data from this date. Finally, in analysing data on Danish‐born individuals who remained in Denmark (to avoid under‐ascertainment of exposure) our results may not be generalizable to migrants.

### 4.4 Clinical and policy implications

These findings confirm to clinicians and policymakers that bereavement by the suicide of a first‐degree relative, including former/current partners, is a risk factor for suicide, supporting its inclusion in suicide prevention strategies as a focus for intervention (termed postvention).^38^ This reinforces survey evidence that suicide exposure in non‐relatives is also of public health importance given impacts on mental health and suicide‐related outcomes.^4^, ^6^, ^7^, ^39^ These findings, and the high prevalence of suicide bereavement,^1^ suggest that clinicians conducting routine psychiatric assessment should broaden their screening for a family history of suicide by inquiring about suicide in other close contacts.

Our PAF and AFe estimates are useful tools for policymakers in considering the relative contribution of suicide bereavement among suicide risk factors. Such figures are illustrative, assuming causality (and no bias or residual confounding), and set out the challenge that if we could prevent suicide bereavement (or address the mediators of suicide risk in suicide‐bereaved relatives), we could prevent 0.69% of suicides in this population. Suicide bereavement is theoretically a modifiable exposure through wider efforts to prevent suicide. However, given these challenges, it may also be possible to reduce the incidence of suicide in the suicide‐bereaved by addressing (at the population or individual level) modifiable mediators of the association between suicide bereavement and suicide. As these mediators have not yet been identified, it is assumed that this could be achieved through investment in a broad range of bereavement support services to address the unmet needs of the suicide‐bereaved.^40^ Evidence to support the effectiveness of postvention in reducing suicide‐related outcomes is lacking, even if it does support effectiveness in reducing outcomes such as depression and anxiety.^41^ These are all candidate mediators of increased suicide risk in suicide‐bereaved relatives, alongside shared social adversity, stigma, shame, loneliness and suicide suggestion.^2^, ^42^

## 5 CONCLUSIONS

In conclusion, these first estimates of the population burden of suicide mortality after suicide bereavement of a first‐degree relative, and the burden specific to the suicide‐bereaved, highlight the importance of reducing exposure to suicide and addressing the known adverse effects of suicide bereavement in care planning as part of global efforts to reduce suicide mortality.

## CONFLICT OF INTEREST

The authors declare that there is no conflict of interest that could be perceived as prejudicing the impartiality of the research reported.

### PEER REVIEW

The peer review history for this article is available at https://publons.com/publon/10.1111/acps.13493.

## ETHICS STATEMENT

The study was approved by the Danish Data Protection Agency and informed consent was waived. Institutional approval to analyse extracted data was provided by the UCL Research Ethics Committee (approval number 14075/001).

## Supporting information


**Appendix S1** Supporting Information.

## Data Availability

Danish registry data are available to researchers with appropriate affiliations on formal application to Statistics Denmark: https://www.dst.dk/en/TilSalg/Forskningsservice
