# Supplementary material for: Proportion of suicides in Denmark attributable to bereavement by the suicide of a first‐degree relative or partner: Nested case–control study
Source: Acta Psychiatr Scand. 2022 Sep 13;146(6):529–39. doi: 10.1111/acps.13493 (PMC9826113; doi:10.1111/acps.13493)
Supplement: Supplementary file 1 — Appendix S1 Supporting Information. [file ACPS-146-529-s001.docx]

# Supplemental methods

**Explanation of the Danish registers’ definition of a couple (whether married or cohabiting), as provided in the Danish Civil Register by** **Statistics Denmark**

variable: CTYPE

Labelled: C family type

<https://www.dst.dk/da/Statistik/dokumentation/Times/cpr-oplysninger/c-type>

- From the value options below:
  a legally married/registered couple are defined as options 11, 12
- a cohabiting couple are defined as options 31, 32, 41, 42

Note that the definition of cohabitees using values 41 and 42 under-estimates the number of same-sex cohabiting couples due to the high likelihood of misclassification of same-sex roommates as couples.

Value options:

01 Single, without children under 18 years living at home

02 Single, with children under 18 years living at home

11 Married couple (different and same sex; civil marriage or religious marriage) without children under 18 years living at home

12 Married couple (different and same sex; civil marriage or religious marriage) with children under 18 years living at home

31 Cohabiting couple (identified through record of joint children via linkage of children to parents’ respective identification numbers) without children under 18 years living at home

32 Cohabiting couple (identified through record of joint children via linkage of children to parents’ respective identification numbers) with children under 18 years living at home

41 Cohabiting couple (different sex, age difference under 15 years, no indication of being genetically related, only two adults in the household aged 16+ years), without children under 18 years living at home

42 Cohabiting couple (different sex, age difference under 15 years, no indication of being genetically related, only two adults in the household aged 16+ years), with children under 18 years living at home

53 non-resident child/children under 18 years

Further notes:

Single: a person who does not live in a couple and who is not a child.

Child: a person under 18 years of age, who does not have children living at home, and who is neither cohabiting nor living together.

Note: A child under the age of 18 is also considered to be a child who does not live with either parent, but with a person who has been married to one of the parents. However, this is conditional on the step-parent not having remarried, as in that case the connection cannot be made between a child and the stepfather or stepmother.

A C-family can only consist of 2 generations, of which the youngest generation must consist of one or more children.

## Timing of covariates

Given the importance of measuring all time-varying covariates in the same set (one case; four controls) at the same timepoint, we used the following approach. For sets in which one person was exposed, covariates for all members of the set were measured on that date of exposure. For sets in which more than one individual was exposed, covariates for all members of the set were measured on the date of exposure of the earliest person exposed. For sets in which no individual was exposed, we assigned all those in the group a pseudo index date, randomly selected from any point between the earliest possible date of exposure (i.e. the earliest date when all five were alive, often 1980) and the latest date of exposure for that set (i.e. the day before the matching date). As the proportion of missing data on income and marital status for the year 1980 was much higher than subsequent years (as this was the year of inception of the Registry of Social Pension and Income) we set 01.01.81 as the start of our eligible period. An exception was where a case within that set had died in 1980, in which case covariates were measured in 1980.

## Linkage issues for individuals born before 1953

Within the Danish registries only individuals born in 1953 or later are linked to their parents and siblings. This is because the Civil Registration System (CRS) was introduced in 1968, at which point these individuals would have been 16 or older when their personal identification number was allocated through the CRS (10). Anyone who was 15 or younger in 1968 would have been living with their legal parents and would not have been allocated a CRS until the age of 16. These individuals would therefore remain unlinked to their parents, and unlinked to any siblings, as linkage to siblings is through shared parental personal identification number. As well as being unlinked to parents and/or siblings, individual born before 1953 who married, cohabited, were widowed, or had children before 1968 would not have been linked to those respective partners or offspring. This explanation accounts for an unknown proportion of the 21% in our sample who were recorded as having had no relatives.

Supplemental Results

Table S1. Classifications according to International Classification of Disease 8^th^ [ICD-8] and 10^th^ [ICD-10] Revisions

| **Category** | **ICD-8 (1969-1993)** | **ICD-10 (1994-2016)^a^** | **Population register** |
| --- | --- | --- | --- |
| **Causes of Death** |  |  | Register of Causes of Death |
| Suicide | E950-E959 or where or where manner of death was recorded as ‘suicide’ | X60-X84 or where or where manner of death was recorded as ‘suicide’ |  |
| Other death | Any death recorded in the Causes of Death Registry except suicide | Any death recorded in the Causes of Death Registry except suicide |  |
| **Self-harm^b^** | E950-E959 or where ‘reason for contact’ was listed as self-harm | X60-X84 or where ‘reason for contact’ was listed as self-harm | Psychiatric Central Research Register; National Patient Register |
| **Psychiatric Disorders^b^** |  |  | Psychiatric Central Research Register |
| PTSD | 309.81 | F43.1 |  |
| Depression | 296.09, 296.2, 298.0, 300.4 | F32.0-F32.2, F32.8-F32.9, F33.0-F33.2, F33.4-F33.9 |  |
| Anxiety | 300.0, 300.2 | F40, F41 |  |
| Substance use | 291, 303, 304 | F10-F19 (excluding F1x.0) |  |
| Severe mental illness | 295, 296.89, 298.29-298.99, 299, 301.83, 296.1, 296.3, 298.1, 296.0 (excluding 296.09) | F20-F29, F30-F31, F32.3, F33.3 |  |
| **Physical Disorders^b^** |  |  | National Hospital Register |
| Cardiovascular disease | 413, 410, 420, 425, 427.91, 427.93, 427.94, 427.09, 427.10, 427.11, 427.19, 427.99 | I20-I22, I30, I42, I44, I46.0, I47.2, I48, I50 |  |
| COPD | 491-492, 518 | J41-J44, J47 |  |
| Diabetes Mellitus | 250 | E105, E109, E111, E115, E119, E131, E135, E139, E141, E145, E149 |  |
| Hypertension | 400-404 | I10-I13, I15 |  |

PTSD=post-traumatic stress disorder; COPD=chronic pulmonary obstructive disease.

^a^ Lowercase x denotes all possible values within the specified digit’s diagnostic category

**^b^** Applies to codes/diagnoses recorded on inpatient admissions. Note that ICD-9 was never implemented in Denmark, hence the transition from ICD-8 to ICD-10

Supplemental Table S2. Differences in characteristics of the sample included and excluded from the main analysis due to missing data

|  | **Complete data**  **(analytic sample)** | | | | **Missing data**  **(excluded sample)** | | | | **Test statistic (df),**  **p-value^b^** |
| --- | --- | --- | --- | --- | --- | --- | --- | --- | --- |
| **Characteristic^a^** | **N** | | **%** | | **N** | | **%** | |  |
| **Total** | 147,154 | | 91.3 | | 14,086 | | 8.7 | |  |
| **Sex** |  | |  | |  | |  | |  |
| Male | 76,831 | | 52.2 | | 7,077 | | 50.2 | | 19.99 (1), <0.001 |
| Female | 70,323 | | 47.8 | | 7,009 | | 49.8 | |  |
| **Age at time of matching (median, IQR)** | 52 | | 40-66 | | 53 | | 36-69 | | 0.60, 0.549 |
| **Age at exposure (median, IQR)** | 46 | | 33-60 | | 51 | | 34-64 | | -15.93, <0.001 |
|  | **Exposed(N=17,991)** | | **Unexposed(N=129,163)** | | **Exposed (N=1,405)** | | **Unexposed(N=12,681)** | |  |
|  | **N** | **%** | **N** | **%** | **N** | **%** | **N** | **%** |  |
| **Sex** |  |  |  |  |  |  |  |  |  |
| Male | 7,301 | 40.6 | 69,530 | 53.8 | 490 | 34.9 | 6,587 | 51.9 | 19.99 (1), <0.001 |
| Female | 10,690 | 59.4 | 59,633 | 46.2 | 915 | 65.1 | 6,094 | 48.1 |  |
| **Age at time of matching (median, IQR)** | 68 | 49-79 | 51 | 39-64 | 74 | 61-82 | 51 | 35-66 | 0.60, 0.549 |
| **Age at exposure (median, IQR)** | 58 | 39-68 | 45 | 32-58 | 61 | 52-69 | 49 | 33-63 | -15.93, <0.001 |
| **Household income level (quartiles)** |  |  |  |  |  |  |  |  |  |
| 1 (lowest) | 2,222 | 12.4 | 14,413 | 11.2 | 10 | 0.7 | 355 | 2.8 | 224.06 (3), <0.001 |
| 2 | 7,073 | 39.3 | 34,264 | 26.6 | 9 | 0.6 | 299 | 2.4 |  |
| 3 | 4,943 | 27.5 | 38,693 | 30.0 | 14 | 1.0 | 430 | 3.4 |  |
| 4 (highest) | 3,753 | 20.9 | 41,793 | 32.4 | 4 | 0.3 | 565 | 4.5 |  |
| Unknown^c^ | 0 | 0.0 | 0 | 0.0 | 1368 | 97.4 | 11,032 | 87.0 |  |
| **Marital status** ^d^ |  |  |  |  |  |  |  |  |  |
| Never married | 3,731 | 20.7 | 37,036 | 28.7 | 157 | 11.2 | 2,745 | 21.7 | 999.04 (3), <0.001 |
| Married/registered partnership | 12,700 | 70.6 | 73,607 | 57.0 | 1,184 | 84.3 | 6,640 | 52.4 |  |
| Divorced/separated | 1,347 | 7.5 | 11,687 | 9.1 | 33 | 2.4 | 925 | 7.3 |  |
| Widowed/bereaved | 213 | 1.2 | 6,833 | 5.3 | 22 | 1.6 | 1,395 | 11.0 |  |
| Unknown^c^ | 0 | 0.0 | 0 | 0.0 | 9 | 0.6 | 976 | 7.7 |  |
| **History of self-harm** | 485 | 2.7 | 2,670 | 2.1 | 9 | 0.6 | 205 | 1.6 | 24.53 (1), <0.001 |
| **History of psychiatric disorder** |  |  |  |  |  |  |  |  |  |
| Any | 1,300 | 7.2 | 9,290 | 7.2 | 69 | 4.9 | 1,02 | 7.9 | 3.17 (1), 0.075 |
| PTSD | 3 | <0.1 | 21 | <0.1 | 0 | 0 | <3 | <0.1 | 0.04 (1), 0.850 |
| Depression | 540 | 3.0 | 3,519 | 2.7 | 43 | 3.1 | 487 | 3.8 | 46.89 (1), <0.001 |
| Anxiety | 99 | 0.6 | 742 | 0.6 | 6 | 0.4 | 72 | 0.6 | 0.07 (1), 0.789 |
| Substance/alcohol use | 684 | 3.8 | 4,806 | 3.7 | 24 | 1.7 | 466 | 3.7 | 2.29 (1), 0.130 |
| Severe mental illness | 352 | 2.0 | 2,910 | 2.3 | 16 | 1.1 | 286 | 2.3 | 0.31 (1), 0.575 |
| **History of physical disorder** |  |  |  |  |  |  |  |  |  |
| Any | 957 | 5.3 | 4,807 | 3.7 | 32 | 2.3 | 324 | 2.6 | 67.99 (1), <0.001 |
| Cardiovascular disease | 557 | 3.1 | 2,573 | 2.0 | 12 | 0.9 | 150 | 1.2 | 61.35 (1), <0.001 |
| Hypertension | 155 | 0.9 | 882 | 0.7 | 8 | 0.6 | 47 | 0.4 | 18.87 (1), <0.001 |
| Diabetes mellitus | 164 | 0.9 | 1,033 | 0.8 | 7 | 0.5 | 77 | 0.6 | 6.46 (1), 0.011 |
| COPD | 144 | 0.8 | 678 | 0.5 | 5 | 0.4 | 66 | 0.5 | 0.69 (1), 0.405 |
| **Kinship to the deceased** |  |  |  |  |  |  |  |  |  |
| Blood relative | 818 | 4.6 | - | - | 27 | 1.9 | - | - | 21.55 (1), <0.001 |
| Partners and step-relatives | 17,173 | 95.5 | - | - | 1,378 | 98.1 | - | - |  |
| **Relationship to the deceased** |  |  |  |  |  |  |  |  |  |
| Child | 785 | 4.4 | - | - | 41 | 2.9 | - | - | 121.99 (3), <0.001 |
| Parent | 3,194 | 17.8 | - | - | 100 | 7.1 | - | - |  |
| Partner | 13,662 | 75.9 | - | - | 1,244 | 88.5 | - | - |  |
| Sibling | 350 | 2.0 | - | - | 20 | 1.4 | - | - |  |
| **Living with deceased at time of death** |  |  |  |  |  |  |  |  |  |
| Yes | 11,826 | 65.7 | - | - | 1,284 | 91.4 | - | - | 399.62 (2), <0.001 |
| No | 5,673 | 31.5 | - | - | 95 | 6.8 | - | - |  |
| Unknown | 492 | 2.7 |  |  | 26 | 1.9 |  |  |  |
| **Time elapsed since split for ex-partners (median, IQR)** | 5 | 2-12 | - | - | 4 | 1-10 | - | - | 1.17, 0.243 |
| **Bereavement status** |  |  |  |  |  |  |  |  |  |
| Suicide bereaved | 844 | 4.7 | - | - | 66 | 4.7 | - | - | 0.00 (1), 0.991 |
| Other bereaved | 17,147 | 95.3 | - | - | 1,339 | 95.3 | - | - |  |
| **Bereavement year/matching year (median, IQR)** ^d^ | 2002 | 1993-2010 | 1991 | 1986-2001 | 1991 | 1985-2000 | 1980 | 1980-1984 | 125.97, 0.001 |
| **Cases (suicide)** ^e^ | 4,818 | 26.8 | 24,695 | 19.1 | 361 | 25.7 | 2,374 | 18.7 | 3.29 (1), 0.070 |

IQR: interquartile range; PTSD: post-traumatic stress disorder; COPD: chronic pulmonary obstructive disease.

Variables derived from Danish population registries are as per Table 1 in main manuscript.

^a^ Values are frequencies and percentages unless otherwise specified.

^b^ Chi-squared test for categorical data; Mann-Whitney U test for continuous data

^c^ Row excluded from test

^d^ Bereavement year in the exposed (bereaved by suicide or other bereavement), matching year in the unexposed.

^e^ based on the following codes in the Register of Causes of Death: ICD-8 codes: E950-E959 or where or where manner of death was recorded as ‘suicide’; ICD-10 codes: X60-X84 or where or where manner of death was recorded as ‘suicide’.

Supplemental Table S3. Risk of suicide in suicide-bereaved individuals compared with two comparison groups (other bereaved and non-bereaved) based on the multiply imputed sample

|  |  | **Unadjusted** | | **Adjusted 1^a^** | | **Adjusted 2^b^** | |
| --- | --- | --- | --- | --- | --- | --- | --- |
| **Reference Group** | **Case/Control** | **OR** | **95% CI** | **OR** | **95% CI** | **OR** | **95% CI** |
| **Non-bereaved as reference category** |  |  |  |  |  |  |  |
| Non-bereaved | 27,069/114,775 | 1.00 | - | 1.00 | - | 1.00 | - |
| Suicide-bereaved | 363/547 | 2.94 | 2.56-3.36 | 3.18 | 2.74-3.69 | 2.90 | 2.49-3.40 |
| **Other bereaved as reference category** |  |  |  |  |  |  |  |
| Other bereaved | 4,816/13,670 | 1.00 | - | 1.00 | - | 1.00 | - |
| Suicide-bereaved | 363/547 | 1.80 | 1.57-2.06 | 1.55 | 1.33-1.80 | 1.45 | 1.24-1.71 |

OR: odds ratio; CI: confidence interval

^a^ Adjusted for sex, marital status, family size, household income level

^b^ Adjusted for all variables in adjustment 1, plus pre-bereavement history of self-harm, mental, and physical health conditions

Supplemental Table S4. Risk of suicide in suicide-bereaved individuals compared with two comparison groups (other bereaved and non-bereaved), stratified by sex, based on the multiply imputed sample.

|  |  |  | **Unadjusted** | | | **Adjusted 1^a^** | | | **Adjusted 2^b^** | | |
| --- | --- | --- | --- | --- | --- | --- | --- | --- | --- | --- | --- |
| **Reference Group** | **Stratum** | **Case/Control** | **OR** | **95% CI** | **p-value for interaction test^c^** | **OR** | **95% CI** | **p-value for interaction test^c^** | **OR** | **95% CI** | **p-value for interaction test^c^** |
| **Non-bereaved as reference category** |  |  |  |  |  |  |  |  |  |  |  |
| Non-bereaved **^d^** | Male | 18,695/57,422 | 1.00 | - | - | 1.00 | - | - | 1.00 | - | - |
|  | Female | 8,374/57,353 | 1.00 | - | - | 1.00 | - | - | 1.00 | - | - |
| Suicide bereaved^e^ | Male | 224/218 | 3.37 | 2.79-4.07 | 0.519 | 3.24 | 2.64-3.97 | 0.780 | 3.27 | 2.64-4.05 | 0.106 |
|  | Female | 139/329 | 3.08 | 2.52-3.76 |  | 3.11 | 2.51-3.85 |  | 2.52 | 2.00-3.19 |  |
| **Other bereaved as reference category** |  |  |  |  |  |  |  |  |  |  |  |
| Other bereaved **^f^** | Male | 2,836/4,513 | 1.00 | - | - | 1.00 | - | - | 1.00 | - | - |
|  | Female | 1,980/9,157 | 1.00 | - | - | 1.00 | - | - | 1.00 | - | - |
| Suicide bereaved^e^ | Male | 224/218 | 1.59 | 1.31-1.93 | 0.197 | 1.40 | 1.14-1.72 | 0.180 | 1.46 | 1.17-1.81 | 0.929 |
|  | Female | 139/329 | 1.92 | 1.56-2.36 |  | 1.72 | 1.38-2.13 |  | 1.44 | 1.13-1.82 |  |

OR: odds ratio; CI: confidence interval`

^a^ Adjusted for sex, marital status, family size, household income level

^b^ Adjusted for all variables in adjustment 1, plus pre-bereavement history of self-harm, mental, and physical health conditions

**^c^** p-value from Wald test for interaction between exposure and sex
